# Effects of Sublingual Duster Mite Drops on Lung Function and Exhaled Nitric Oxide in Children with Multiple and Single Allergic Respiratory Diseases

**DOI:** 10.1155/2020/7430936

**Published:** 2020-11-04

**Authors:** Li Wang, Tao Ai, Ronghua Luo, Yinghong Fan, Huiling Liao, Wanmin Xia, Cheng Xie, Yaping Duan, Yanru Liu

**Affiliations:** Pediatric Respiratory Medicine Department, Chengdu Women's and Children's Central Hospital, School of Medicine, University of Electronic Science and Technology of China, Chengdu 611731, China

## Abstract

**Objective:**

To evaluate the efficiency of sublingual immunotherapy with Dermatophagoides Farinae Drops in children with single and multiple respiratory allergic diseases.

**Methods:**

Seventy-one children with allergic respiratory diseases who had been treated with Dermatophagoides Farinae Drops for one year or more were divided into a single allergic group (12 cases) and multiple allergic group (59 cases). The rhinitis score, daytime and night symptom score of asthma, VAS score, drug score, pulmonary function, and FeNO level before and after treatment were evaluated and compared between the two groups.

**Results:**

The rhinitis score, night symptom score, VAS score, and drug score in the single allergic group after treatment were significantly lower than those before treatment (*p* < 0.05), but there was no significant difference in the daytime symptom score before and after treatment (*p* > 0.05). The rhinitis score, VAS score, and drug score in the multiple allergic group after treatment were significantly lower than those before treatment (*p* < 0.05), but there was no significant difference in the scores of daytime symptoms and nighttime symptoms before and after treatment (*p* > 0.05). In both the single allergic group and multiple allergic group, the pulmonary function indexes of the patients were significantly improved after treatment, and the FeNO after treatment was significantly lower than that before treatment (*p* < 0.05). There was no significant difference in scores, pulmonary function, and FeNO between the two groups (*p* > 0.05).

**Conclusion:**

Sublingual specific immunotherapy is effective in treating multiple and single allergic respiratory diseases in children.

## 1. Introduction

Respiratory allergic diseases are one of the most common diseases in pediatrics, including allergic asthma (AS), allergic rhinitis (AR), and cough variant asthma; among them, AS and AR are the most common respiratory allergic diseases in childhood [1]. In China, the prevalence of AR in children has increased from 9.1% in 2001 [2] to 15.4% in 2010 [3], and house dust mites have been documented to be the most prevalent allergens [4]. Symptomatic treatment is based on antihistamines and corticosteroids. Allergic asthma is characterized by chronic inflammation which results in recurrent attacks of cough, wheezing, sometimes chest tightness, and variable airflow obstruction [5, 6]. It is a major public health problem affecting over 300 million people worldwide. According to the latest position papers, allergen specific immunotherapy has practically no controversies in the treatment of AR and allergic asthma [7]. Also, in the latest national guidelines for the diagnosis and treatment of AR, it is suggested that allergen-specific immunotherapy should be used as first-line treatment for AR [8]. In the recent years, the incidence rate of allergic diseases in children has been increasing, which has caused great harm to children's physical and mental health, including a heavy economy burden on the family [9].

Dermatophagoides Farinae Drops, a sublingual immunotherapy drug, has been shown to be effective and safe in preschool and school-age patients with house dust mites- (HDMs-) induced AR and adult patients with AA [10, 11]. Though the effectiveness of sublingual specific immunotherapy (SLIT) has been reported [12], there are relatively few data about the efficacy of sublingual Dermatophagoides Farinae Drops in the treatment of multiple and single respiratory allergic diseases in children. Therefore, this study evaluated the efficacy of sublingual Dermatophagoides Farinae Drops in children with multiple and single allergen respiratory allergic diseases.

## 2. Methods

### 2.1. Patients

From February 2016 to February 2018, 80 children diagnosed with asthma accompanied with rhinitis or not, who were treated with Dermatophagoides Farinae Drops, were selected. According to the skin prick test results, they were divided into two groups: ① the single allergic group (20 cases): the children only showed positive for acarid dust or house dust mite. ② The multiple allergy group (60 cases): 60 children were positive for, at least, one allergen besides mite.

The inclusion criteria were the following: ① AS according to Guidelines for the Prevention and Treatment of AS in Children (2016) and Guidelines for Diagnosis and Treatment of AS in Children (Chongqing, 2010) [13, 14]; ② the positive dust mite prick test with or without other allergens; and ③ nonacute asthma. The exclusion criteria were: ① acute asthma (FEV1 less than 70% of predicted value); ② severe allergic reaction.

### 2.2. Treatment

The sublingual Dermatophagoides Farinae Drops (S20060012) produced by Zhejiang Wowu Biotechnology Co., Ltd. were divided into 1–4 bottles, corresponding to the total protein concentration of 1, 10, 100, and 333 *μ*g/ml, respectively.

The drug is kept under the tongue for 1–3 minutes before swallowing. The treatment includes an induction and a maintenance phase: the increasing period is one week for each bottle 1, 2, and 3 and from the first day to the seventh day of each week includes 1, 2, 3, 4, 6, 8, and 10 drops. During the maintenance period, Dermatophagoides Farinae Drops of bottles 4 were used and 3 drops/day were maintained until the end of the treatment. During the treatment of SLIT, other drugs for asthma and rhinitis were used according to the clinical symptoms of the children according to the guidelines [6, 15].

### 2.3. Pulmonary Function

The pulmonary function of children was evaluated by using the instrument from Jaeger (Master Screen, Jaeger, Germany). The measured parameters include forced vital capacity (FVC), forced expiratory volume in one second (FEV1), forced expiratory volume in one second (FEV1/FVC), maximum forced expiratory peak flow (PEF), forced expiratory volume in 25%, 50%, and 75% of vital capacity (FEF25, FEF50, FEF75), and maximum mid expiratory flow (MMEF) [16].

### 2.4. Determination of Nitric Oxide

The exhaled nitric oxide was measured by using the exhaled nitric oxide tester (NIOX, MINO, Sweden). The level of FeNO was measured according to the standard measurement guide recommended by the American Thoracic Society/European Respiratory Society (ATS/ESR) [17].

### 2.5. Evaluation Indicators

The severity of rhinitis symptoms was evaluated with the “Four-point method” (Table 1) [18]. The symptom score of asthma includes the daytime symptom score, nighttime symptom score, and VAS score, which are evaluated according to the severity of symptoms and the impact on life (Table 2) [19].

The drug score was used to evaluate the application of drugs, which is mainly used to record the use of drugs pointing to symptoms in children to evaluate the clinical efficacy of immunotherapy. The “Three-steps” scoring method was used (Table 3) [14].

### 2.6. Statistical Analysis

SPSS 17.0 software was used to analyze the data. If the quantitative data obey the normal distribution, the mean ± standard deviation ($\bar{X}\pm S$) is used for description, and the *t*-test of two independent samples (Student's test) is used for group comparison. The median, upper, and lower quartiles (m, p25–p75) were used to describe the normal distribution of quantitative data, and the rank sum test of two independent samples was used to compare between groups (Mann–Whitney *U*-test). Qualitative data were described by percentage and compared by the chi square test. To the test level, *α* = 0.05 was used as the inspection standard.

## 3. Results

### 3.1. Basic Characteristics

Eighty children treated with Dermatophagoides Farinae Drops were included. In the single allergic group, 20 patients were enrolled. After one year's follow-up, 6 patients lost the follow-up and voluntarily withdrew. Two patients dropped out from treatment due to the acute asthma (FEV1 less than 70% of the predicted value). Finally, 12 patients completed the study, including 9 males (75.0%) and 3 females (25.0%). In the multiple allergy group, 60 people were enrolled, 1 person was lost to follow-up, and 59 people completed the study. There were 38 (64.4%) males and 21 (35.6%) females, respectively. There was no significant difference in gender composition between the two groups (*χ*2 = 0.139, *p*=0.710). The age of the single sensitization group was 6.5 ± 3.1 years, and that of multiple sensitization group was 7.3 ± 2.9 years. There was no significant difference of age between the two groups (*t* = 0.856, *p*=0.395).

Comparison of the rhinitis score, asthma score, VAS score, and drug score before and after treatment.

The rhinitis score, night symptom score, VAS score, and drug score after treatment in the single allergic group were significantly lower than those before treatment (*p* < 0.05), while there was no significant difference in the daytime symptom score before and after treatment (*p* > 0.05) (Table 4).

The rhinitis score, VAS score, and drug score after treatment in the multiple allergic group were significantly lower than those before treatment (*p* < 0.05), while there was no significant difference in the daytime symptom score and night symptom score before and after treatment (*p* > 0.05) (Table 5).

### 3.2. Comparison of Pulmonary Function and FeNO before and after Treatment

In both the single allergic group and multiple allergic group, the pulmonary function of the patients after treatment was significantly higher than that before treatment, while FeNO after treatment was significantly lower than that before treatment (*p* < 0.05) (Tables 6 and 7).

### 3.3. Comparison of the Two Groups

There was no significant difference of the scoring between the two groups (*p* > 0.05) (Table 8). There were also no significant differences of pulmonary function and FeNO between the two groups (*p* > 0.05) (Table 9).

## 4. Discussion

Allergen-specific immunotherapy (AIT) is considered as the only way to change the natural course of allergic diseases. The World Allergy Organization (WAO) recommends sublingual-specific immunotherapy (SLIT) as the initial and early treatment for allergic diseases and not when drugs are ineffective after the failure of drug treatment [20]. A meta-analysis confirmed the effectiveness of sublingual-specific immunotherapy in 3–18-year-old children with AS [21]. Another meta-analysis also confirmed that sublingual immunotherapy has a significant effect on children with AS and AR caused by dust mite allergy [22]. In this study, we have found that sublingual-specific immunotherapy of Dermatophagoides Farinae Drops is effective in treating multiple and single allergic respiratory diseases in children.

A large cross-sectional multicenter study in China found that more than 90% of allergic patients in China are allergic to two or more allergens, of which 83.7% are allergic to both dust mites and house dust mites [4]. Other studies also showed that the proportion of patients with multiple allergies was much higher than that of patients with single allergies [23]. Specific Dermatophagoides Farinae immunotherapy was effective in children with AS due to either single allergies or multiple allergies [24]. A retrospective study on 124 children with AR and AS who received SLIT treatment for 3 years showed that the sublingual Dermatophagoides Farinae had a similar effect on AR [25]. EAACI guidelines emphasize that sublingual desensitization with a single allergen were equally effective in patients with multiple allergies, which may be due to the cross reaction between different allergens and the inhibitory effect of cytokines on immune response [26].

There are a few studies evaluating the efficacy of sublingual Dermatophagoides Farinae in the treatment of single and multiple allergic respiratory diseases in children. A recent study showed that, after sublingual immunotherapy, the clinical indexes of children with both single and multiple allergies were significantly improved [27]. Another study also found that sublingual Dermatophagoides farinae can significantly improve the nasal symptoms of children with single dust mite allergy and dust mite combined with other allergies and reduce the use of drugs pointing to symptoms; moreover, the effect of Dermatophagoides farinae on children with single and multiple allergies was similar [28].

Our current study found that the rhinitis symptoms, asthma night symptoms, VAS scores, and drug scores were significantly improved in the single allergic group, but the daytime symptoms were not significantly improved after treatment. Rhinitis symptoms, VAS scores, and drug scores were significantly improved in the multiple allergy group, but the daytime symptoms and night symptoms were not significantly improved before and after treatment. Our study also confirmed that the efficacy of sublingual Dermatophagoides farinae drops in the treatment of respiratory allergic diseases is equivalent in children with single and multiple allergies, which was in accordance with a previous study [27].

The measurement of pulmonary function has been one of the important bases for the diagnosis and evaluation of asthma control. FeNO is also one of the most valuable markers reflecting allergic airway inflammation [29]. Therefore, pulmonary function and FeNO can be used as the evaluation indexes of SLIT in the treatment of respiratory allergic diseases. This study showed that the pulmonary function indexes and FeNO levels were significantly improved in both single and multiple allergic groups. It was found that sublingual-specific immunotherapy can improve pulmonary function (PEF, FEV1%) and FeNO in children with AS and AR [30]. SLIT treatment can significantly improve the symptoms, reduce the level of FeNO, reduce airway inflammation, and improve the ventilation index of large and small airways in children with AS and AR [31].

A previous study showed that the sublingual immunotherapy of house dust mite could significantly improve the airflow obstruction (FEV1), significantly reduce the level of FeNO, and reduce the eosinophilic inflammation of the airway in patients with AS and AR [32]. Another study also showed that SLIT could significantly improve the symptoms, reduce the long-term use of drugs, and improve FEV1 [33], which was also in accordance with our results. However, one study including 26 children with AS and AR, treated with SLIT and followed up for 3 years, had no significant improvement in pulmonary function [34]. A retrospective study of 124 children with AS and AR treated with SLIT for 3 years showed that there was no significant difference in the improvement of lung function (PEF, FEV1) of children with single or multiple allergies by SLIT [25]. Therefore, further study with lager sample size was needed to confirm the conclusion. There was no study investigating the difference in the improvement of small airways (FEF25-75, fef75/fef25) and FeNO levels in children with single or multiple allergy treated with SLIT. In this study, it was found that the treatment of sublingual Dermatophagoides Farinae drops could significantly improve the small airway index (FEF25-75, fef75/fef25) and the level of FeNO in the two groups.

In conclusion, our study found that sublingual-specific immunotherapy was effective in treating multiple and single allergic respiratory diseases in children, which further confirmed the clinical use of Dermatophagoides Farinae Drops in respiratory allergic diseases.

## Figures and Tables

**Table 1:** Allergic rhinitis symptom rating scale.

Grading	Sneeze^A^	Shed tears^B^	Antiques with history	Rhinopruritus
1 point	3–5	≤5	Inhale consciously	Interrupted
2 points	6–10	6–9	Intermittent or interactive	Allelopathy of ants, tolerable
3 points	≥11	≥10	Almost all day	Allelopathy of ants, intolerable

Note: ^A^the number of a consecutive sneezes; ^B^the number of daily nasal discharge.

**Table 2:** Evaluation of daytime and night symptoms of asthma.

Grading	Daytime symptom score of asthma	Night symptom score of asthma
0 point	Asymptomatic	Asymptomatic
1 point	Few symptoms and short duration	Wake up once or early
2 points	Short symptoms ≥2 times	Wake up twice, including early
3 points	Many times mild symptoms in day, little impact on life and work	Wake up many times
4 points	Many times serious symptoms in day, impact on life and work	Cannot sleep at night
5 points	The symptoms were so severe that the subjects could not work and live normally	

**Table 3:** Symptomatic drug rating scale.

Grading drug consumption scores	Drugs pointing symptom
1 point	Oral and/or local antihistamines, antileukotrienes, and bronchodilators
2 points	Nasal glucocorticoids/inhaled glucocorticoids
3 points	Oral glucocorticoids^*∗*^
	Combination (glucocorticoids and *β*2-receptor agonist)

^*∗*^Oral glucocorticoids belong to the 3 points group.

**Table 4:** Comparison of scores of the single allergy group before and after treatment.

Variable	Before treatment	After treatment	*Z*	*p*
Rhinitis score	0.5 (0.0, 3.0)	0.0 (0.0, 1.0)	2.220	0.026
Daytime symptom score	0.0 (0.0, 0.5)	0.0 (0.0, 0.0)	1.633	0.102
Night symptom score	2.5 (0.0, 4.0)	0.0 (0.0, 0.0)	2.375	0.018
VAS score	5.0 (3.0, 5.5)	0.5 (0.0, 2.0)	2.940	0.003
Drug score	4.0 (3.0, 5.0)	0.0 (0.0, 2.5)	2.949	0.003

**Table 5:** Comparison of scores in the multiple allergy group before and after treatment.

Variable	Before treatment	After treatment	*Z*	*p*
Rhinitis score	0.0 (0.0, 2.0)	0.0 (0.0, 1.0)	4.520	<0.001
Daytime symptom score	0.0 (0.0, 1.0)	0.0 (0.0, 1.0)	1.448	0.148
Night symptom score	1.0 (0.0, 3.0)	0.0 (0.0, 4.0)	1.024	0.306
VAS score	4.0 (2.0, 6.0)	1.0 (0.0, 2.0)	6.052	<0.001
Drug score	4.0 (3.0, 4.5)	0.0 (0.0, 2.0)	6.571	<0.001

**Table 6:** Comparison of pulmonary function and FeNO in the single allergic group before and after treatment.

Variable	Before treatment	After treatment	*t*	*p*
FVC	90.7 ± 11.7	103.9 ± 12.4	3.804	0.003
FEV1	84.1 ± 11.0	106.7 ± 9.2	8.093	<0.001
PEF	90.2 ± 13.5	108.3 ± 4.0	5.247	<0.001
PEF75	52.2 ± 20.1	94.2 ± 20.7	4.998	<0.001
PEF50	54.6 ± 12.9	94.8 ± 18.9	6.408	<0.001
PEF25	58.6 ± 17.6	98.6 ± 23.0	4.602	0.001
MMEF	52.8 ± 12.9	93.1 ± 21.1	5.080	<0.001
FeNO	39.5 (25.5, 89.0)	18.5 (14.0, 57.5)	3.061	0.002^a^

Note: (a) Mann–Whitney *U*-test of two independent samples.

**Table 7:** Comparison of pulmonary function and FeNO in the multiple allergic group before and after treatment.

Variable	Before treatment	After treatment	*t*	*p*
FVC	89.2 ± 11.4	104.1 ± 12.2	9.761	<0.001
FEV1	89.7 ± 11.3	105.6 ± 13.5	8.997	<0.001
PEF	92.3 ± 13.1	105.7 ± 14.1	6.764	<0.001
PEF75	58.0 ± 18.8	89.5 ± 24.9	9.807	<0.001
PEF50	70.1 ± 14.8	92.4 ± 20.4	8.827	<0.001
PEF25	68.9 ± 20.2	97.2 ± 19.8	8.812	<0.001
MMEF	66.5 ± 16.2	90.9 ± 22.8	9.492	<0.001
FeNO	31.0 (23.5, 44.0)	15.0 (11.0, 23.0)	6.671	<0.001^a^

Note. (a) Mann–Whitney *U*-test of two independent samples.

**Table 8:** Comparison of scores between the single allergy group and multiple allergy group.

Variable	Single allergy group (*n* = 12)	Multiple allergy group (*n* = 59)	*Z*	*p*
Rhinitis score	1 (1, 1)	0 (0, 0)	0.713	0.476
Daytime symptom score	0 (0, 0)	0 (0, 0)	0.499	0.618
Night symptom score	0 (0, 0)	0 (0, 0)	0.792	0.428
VAS score	2 (1, 3)	0 (0, 2)	1.704	0.088
Drug score	0 (0, 2)	0 (0, 2)	0.363	0.717

**Table 9:** Comparison of pulmonary function and FeNO between single allergy groups and multiple allergy groups.

Variable	Single allergy groups (*n* = 12)	Multiple allergy groups (*n* = 59)	*t*	*p*
FVC	97.4 ± 14.5	97.9 ± 11.9	0.113	0.910
FEV1	92.8 ± 14.4	97.4 ± 12.2	1.159	0.250
PEF	99.0 (94.3, 106.5)	97.6 (90.2, 107.2)	0.040	0.968^a^
PEF75	56.3 ± 22.2	60.6 ± 20.2	0.653	0.516
PEF50	69.6 ± 23.3	78.6 ± 18.1	1.498	0.139
PEF25	81.2 ± 21.3	92.7 ± 18.0	1.943	0.056
MMEF	65.8 ± 22.0	75.9 ± 19.6	1.596	0.115
FeNO	18.5 (13.5, 57.8)	15.0 (11.0, 23.0)	1.582	0.114^a^

Note. (a) Mann–Whitney *U*-test of two independent samples.

## Data Availability

The authors agree to share the data and materials of this paper.
